# Climate, Interventions, and Malaria Outcomes in a Warming World: Towards Climate-Smart Malaria Control in Kenya

**DOI:** 10.3390/tropicalmed10120335

**Published:** 2025-11-27

**Authors:** Bryan O. Nyawanda, Eric Ochomo, James D. Otieno, Kibor Keitany, Beatrice K. Machini, Penelope Vounatsou

**Affiliations:** 1 Department of Epidemiology and Public Health, Swiss Tropical and Public Health Institute, Kreuzstrasse 2, CH-4123 Allschwil, Switzerland; 2 Faculty of Science, University of Basel, Petersplatz 1, CH-4001 Basel, Switzerland; 3 Center for Global Health Research, Kenya Medical Research Institute, P.O. Box 1578, Kisumu 40100, Kenya; 4 Vector Department, Liverpool School of Tropical Medicine, Pembroke Place, Liverpool L3 5QA, UK; 5 WHO Kenya Country Office, P.O. Box 45335, Nairobi 00100, Kenya; 6 National Malaria Control Programme, Ministry of Health, P.O. Box 19982, Nairobi 00202, Kenya

**Keywords:** climate change, climate variability, early warning, forecasting, insecticide treated nets, Kenya, malaria, mortality, vector control

## Abstract

Malaria control in sub-Saharan Africa lies at the intersection of changing climate suitability and the scale-up of vector control and case management. Drawing on recent evidence from Kenya, we argue that climate variability already exerts effects on malaria outcomes comparable to, and sometimes stronger than, those of commonly measured interventions at local scales. Transmission dynamics display non-linear, lagged relationships with temperature and rainfall. As a result, climate change is expected to alter prevailing conditions and extremes, reshaping the geography and seasonality of malaria risk. At the same time, socio-economic development and vector control intervention such as insecticide-treated bed nets (ITNs) and timely case management continue to reduce malaria incidence and deaths, especially among young children. However, their population-level impact depends on when and where interventions are deployed relative to climate-favoured windows of transmission. We propose a practical agenda for “climate-smart” malaria control in Kenya advocating for dynamic targeting of interventions according to observed climate lags and thresholds, sustaining protection for the youngest, and innovating approaches for school-age reservoirs of infection. Access to effective care should be re-conceptualized as a climate-adaptation strategy, and short-term, locally tailored forecasts should be embedded into routine planning to support anticipatory and equitable malaria control.

## 1. Introduction

**Figure 1:**
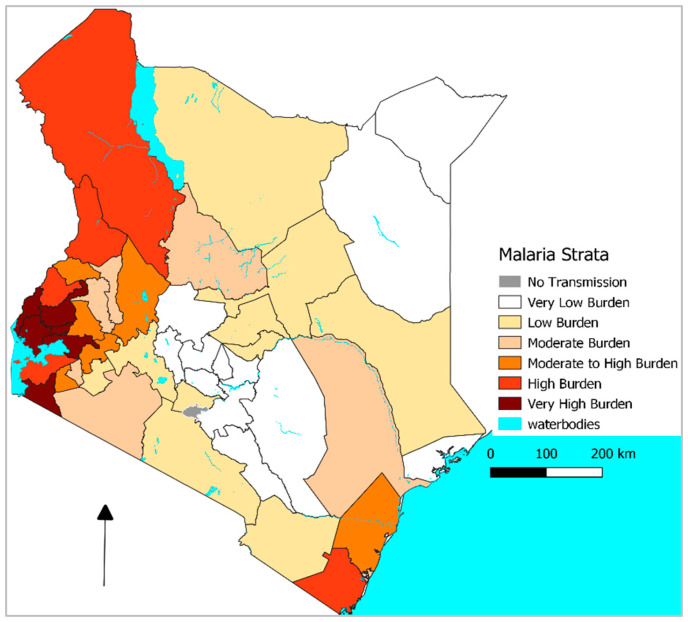
Map showing the current malaria transmission zone classification. Source: National Malaria control Programme, Kenya [6].

After two decades of progress, the downward trends in malaria incidence in parts of Africa have plateaued [1]. In Kenya, transmission remains spatially heterogeneous. Previously, transmission was highest in the lake region in western Kenya and the coastal regions [2]. However, recent data highlight increased incidence in areas previously known for seasonal or epidemic transmission; Figure 1 [3,4,5,6]. Malaria is a climate sensitive disease; its transmission is associated with optimal temperatures, rainfall and humidity levels, together with land use patterns which affect the vector biology [7]. The association is unimodal, very low or very high temperatures and rainfall being unfavourable for vector proliferation [7,8]. The global mean temperature has increased [9] and there is evidence of shifts in rainfall and temperature patterns [10,11]. While earlier studies observed declines in rainfall in East Africa contrary to the predicted increase under climates change [10], recent studies have observed increased rainfall and shifts in temperature patterns [11,12]. These shifts are likely to influence the redistribution of malaria vectors to new areas, including highlands [8]. The shifts have implications on vector control planning due to inadequate resources, requiring innovative ways in maximizing the impact of control interventions [13]. As climate change alters the magnitude, timing, and variability of temperature and rainfall, the National Malaria Control Programme (NMCP) needs to continually recalibrate how control tools such as insecticide-treated nets (ITNs), indoor residual spraying (IRS), vaccination, chemoprevention, case management, and novel control tools such as spatial repellents and attractive targeted sugar baits (ATSB) are distributed across space and time [1,13]. This commentary synthesizes recent studies from Kenya to illuminate (i) national-scale changes in parasite prevalence, (ii) the contributions of climate variability and control interventions to incidence, and (iii) environmental and health system- determinants of under-five malaria mortality. It further translates these insights into a practical framework for climate-smart malaria control.

## 2. Main Text

### What the Recent Evidence Shows

Overall malaria risk in Kenya has decreased in recent years, but not uniformly across space or time [3,5]. Successive Kenya Malaria Indicator Surveys, analyzed using Bayesian geostatistical models, show substantial reductions in parasite prevalence at the national scale, particularly among children under five, alongside persistent or intensifying transmission including in semi-arid northern counties and at the margins of historically lower-risk zones [5,14]. This pattern suggests redistribution of vulnerability rather than a monotonic decline. Recent studies in Kenya’s semi-arid northern counties detected *Anopheles stephensi*, an invasive mosquito vector which is adapted to harsh environmental conditions and capable of transmitting both *Plasmodium falciparum* and *P. vivax*, highlighting the need for heightened surveillance and control [15].

Spatial covariates such as nightlights (a proxy of socioeconomic development and urbanization) and household ITNs/IRS use are consistently associated with lower parasitaemia, highlighting how improved living conditions and preventive tools act together to reduce transmission [16].

Time series and spatio-temporal studies from Kenya show that climate variability exerts large and predictable influences on incidence and mortality due to malaria [17,18,19,20,21]. Typically, rainfall and humidity raise risk after lags of approximately 1–2 months [17,22]. While moderate rainfall creates breeding habitats, heavy rainfall initially washes out larvae, but as the floods recede, they leave new pools that can act as breeding habitats if poorly managed [23]. Temperature influences are also non-linear. In the western lowlands, very high temperatures can suppress transmission, whereas warming in cooler highlands can increase suitability [3]. Empirical dynamic models use observed time series data to reconstruct system dynamics and forecast potential threshold crossing [24]. Using empirical dynamic modelling, recent work moves beyond correlation to infer causality and identify operational thresholds and effective lags for short-term forecasting [12]. Forecast skill is high when appropriate covariate ranges and lags are selected [12], demonstrating that climate thresholds can be operationalized for early warning. At the severe outcome end, village- and catchment-level models link under-five malaria mortality to seasonal rainfall, greenness, and agricultural land cover [20]. On the other hand, shorter travel time to care and increased ITN coverage, elevation, and socioeconomic status are associated with reduced mortality due to malaria [16,20]. These emphasize that while transmission drivers are climate-sensitive, who dies reflects differences in protection and access. Methodologically, Kenya’s evidence base has expanded from cross-sectional survey mapping to decision-ready spatio-temporal intelligence. Routine health-facility test-positivity data, calibrated via Bayesian space–time models, now support micro-stratification with exceedance probabilities to flag within-county hotspots, justifying focal IRS or intensified case management where community surveys are sparse [5].

Taken together, the Kenyan literature converges on three programme implications. First, maintaining national gains while targeting persistent and emerging hotspots using high-resolution risk and uncertainty surfaces rather than broad administrative averages. Second, operationalizing short-lead, climate-aware forecasts to time ITN distributions, IRS rounds, and commodity pre-positioning. Third, pairing climate-sensitive surveillance with improved access, reducing travel time to care, and accelerating diagnosis and treatment, so that unfavourable weather does not translate into avoidable deaths, especially in young children. Together, these strategies form an integrated approach that combines targeted prevention, timely response, and improved healthcare access to minimize malaria risk during adverse climate conditions.

## 3. Mechanisms and Interactions

Malaria dynamics reflect interacting non-linear processes. Vector ecology, parasite development, human behaviour, and health system response jointly shape transmission. Temperature accelerates parasite development and vector biting up to an optimum, beyond which excess heat reduces survival [7]. Rainfall creates breeding sites, while extreme rainfall can initially flush larvae, before creating pools that support larvae development [23]. Humidity supports adult survival, and wind speed affects mosquito flight and dispersal [12]. These climate sensitivities typically manifest with lags of weeks to months.

**Figure 2:**
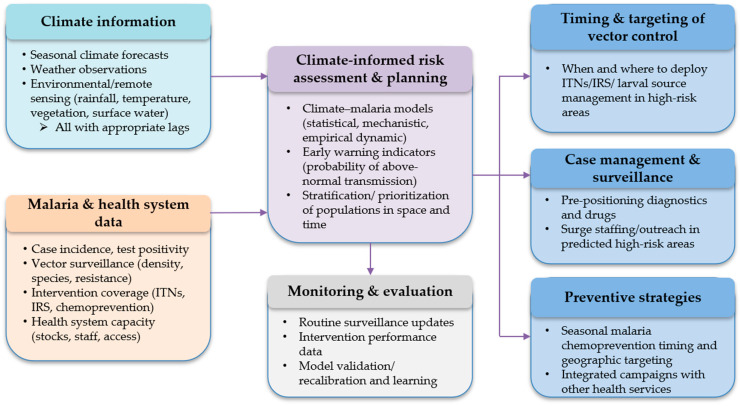
A schematic diagram illustrating the climate-smart malaria control framework.

Interventions modulate climate-driven potential. ITNs reduce human–vector contact while IRS reduces indoor vector survival. Chemoprevention and prompt case management shorten infectious periods. The population level impact of these tools depends on coverage, adherence, utilization, and timing relative to climate-favoured windows. If a net distribution misses the pre-transmission buildup, or if access constraints delay treatment during rainy season spikes, the benefit may decrease [13,21]. Conversely, delivering interventions ahead of forecasted transmission pulses yields larger gains, especially for children under five, who bear the highest mortality risk. Figure 2 provides a schematic diagram where climate and environmental information are combined with malaria and health system data to support climate-informed risk assessment and planning, which in turn guides the timing, targeting, and choice of malaria control interventions. Monitoring and evaluation feed back into the system, updating data streams and improving future risk assessments and operational decisions.

## 4. Policy and Practice: A Climate-Smart Agenda for Kenya

A climate-smart programme prioritizes places where climate pressure is high and intervention coverage is sub-optimal. The semi-arid northern counties, where prevalence declines have stalled or reversed, and the invasive *An. stephensi* vector has been detected, warrant flexible, climate-informed targeting. In these settings, surveillance, pre-season ITN top-ups, focal IRS where vectors rest indoors, and surge case-management capacity should be deployed when sub-seasonal and seasonal forecasts indicate elevated risk. These actions align resources with where and when transmission is most likely to intensify.

Operational planning should be organized around empirically observed lags and thresholds, not long-run averages. Kenyan time series evidence indicates that rainfall and humidity influence incidence after roughly 2 months, and temperature at about 1 month, particularly in western Kenya [12,25]. Transmission peaks within suitability ranges and can decline once temperatures consistently exceed 35 °C [26]. Translating these into simple decision rules, such as pre-position commodities when 1-month land surface temperature is within 30–35 °C and 2-month rainfall is in the upper tercile, yields more reliable triggers than generic “hot/wet” labels. These relationships may differ by region and as such, area-specific models are necessary.

Although risk and harm are not distributed evenly across age groups, protection should be sustained for the youngest while addressing school-age reservoirs. Bed-net protection remains strongest for children under-five and pregnant women; therefore, maintaining high coverage and consistent use in these groups is essential. However, school-age children carry the highest parasite prevalence and sustain transmission [3,27]. Programmes should consider school-based screening and chemoprevention, behaviour-informed vector control around schools and households, and community health education aligned with forecasted transmission pulses as control measures for this group and the wider population, particularly in moderate- to high-transmission areas.

Access to effective care should be treated explicitly as climate adaptation. Travel time to health facilities is a robust predictor of under-five malaria mortality in Kenya [20,28]. As rains cluster risk in space and time, positioning community health workers, rapid diagnostic tests, and artemisinin-based combination therapy stocks in hard-to-reach areas before peak transmission can prevent avoidable deaths. In practice, universal, rapid access to diagnosis and treatment converts climate-loaded transmission potential into managed clinical demand, reducing mortality even when incidence spikes.

Programmes should embed local forecasts into routine planning and leverage socioeconomic signals to prioritize and monitor interventions. Once local drivers, lags, and thresholds are understood, short-term prediction becomes accurate enough to guide rolling quarterly or semi-annual updates to supply chains, staffing, and risk communication. In parallel, proxies such as nightlights can help identify areas where structural improvements are translating into lower risk and where they are not, supporting micro-planning and equity audits alongside conventional coverage metrics.

## 5. Limitations of the Current Evidence

Findings are Kenya-specific and may not generalize to other ecologies or health systems without adaptation. Facility time series capture care-seeking as well as true incidence and can be affected by exogenous shocks such as elections, strikes and stock-outs, biasing short-term signals. Forecast skill is data-dependent and decays with data gaps, changes in diagnostics/reporting, or shifting catchments, and therefore requires regular retraining and recalibration to accommodate changes. Lastly, climate variability (seasonal and interannual) is not synonymous with climate change; relationships identified over short periods should not be extrapolated across decades without explicit climate-scenario modelling, and attention to non-stationarity in vectors, parasites, interventions, and human behaviour.

## 6. Conclusions

Kenya, like other African countries has made tremendous progress in malaria control. Programmes can be smarter by aligning interventions with where risk concentrates and when climate favours transmission. High-resolution risk maps, routine micro-stratification, and short-term, climate-aware forecasts make it possible to pre-empt surges, allowing ITN top-ups, focal IRS, and case management to be timed to the season rather than the calendar. Protecting children under five years of age should remain a priority, while addressing school-age reservoirs to suppress onward transmission. Treating access to care as adaptation and pre-positioning case management and vector control tools in hard-to-reach areas ahead of rains will convert climate volatility into managed clinical demand, averting deaths and shifting Kenya from reactive control to anticipatory, precision public health.

## Data Availability

No new data were created or analyzed in this study. Data sharing is not applicable to this article.
